# Municipal amalgamations and the quality of public services: A study based on city-county mergers in China

**DOI:** 10.1371/journal.pone.0272430

**Published:** 2022-08-08

**Authors:** Zijun Mao, Deqi Wang, Guoping Zhang

**Affiliations:** 1 School of Urban Economics and Public Administration, Capital University of Economics and Business, Beijing, China; 2 China Development Institute, Shenzhen, China; Newcastle University Business School, UNITED KINGDOM

## Abstract

Municipal amalgamation is one of the core policy tools for Chinese government intervention in urbanization. The city-county merger policy provides a valuable research object for examining whether government-led urban expansion improves the quality of public services. By using city panel data from 2003 to 2019, this paper examines the policy effects of city-county mergers on the quality of public services using the Propensity Score Matching-Difference-in-Differences (PSM-DID) model. The results indicate that, after controlling for other factors, city-county mergers have increased the quality of public services by 1.2%. A placebo test has validated the robustness of this positive effect. Through further tests, the paper finds that the policy has positively affected all three aspects of the quality of public services in China: education, health care, and transport infrastructure, with the greatest impact being on education. Using a case study of a city-county merger in the Fenghua District of Ningbo, this paper depicts the transmission mechanism and argues that the policy affects the quality of public services by providing institutional security (financial and administrative power) and promoting regional integration in the new city area.

## Introduction

Local governments have undergone a series of reforms in most industrialized and urbanized countries since the Second World War. The re-drawing of municipal boundaries is considered to be the most essential and radical reform [1], and must be viewed as an intrinsic moment of the current wave of globalization [2]. Many European countries, such as the United Kingdom, Germany, and Sweden, have favored municipal amalgamations to enable scale effects and improve urban management by increasing the size of municipalities over the past decades [3–6].

In academic circles, scholars have taken positions both in favor of and against the implementation of municipal amalgamations. On the one hand, a common argument favoring amalgamating municipalities has relied on its ability to harvest scale effects and reduce administrative costs [7–9]. On the other hand, the negative viewpoint has advocated that the amalgamation effect is insignificant to local public expenditures and taxation [10,11] and that a centralized local government structure after consolidation implies a loss of efficiency because of heterogeneous preferences among local governments [12]. Some scholars have even argued that territoriality, and even geography itself, gets dissolved as the accelerated process of globalization [13] and national borders have become irrelevant, redundant, or obsolete in modern capitalist states [14]. In addition to these diverging attitudes, a neutral attitude has been held by a number of scholars, arguing that the optimal jurisdiction size is determined by balancing between scale economics, inter-jurisdictional spillovers, and efficiency losses from heterogeneous preferences [5,15].

The studies above have provided a rich perspective on the effects of municipal amalgamations; however, the literature has focused on experiences from highly industrialized countries where territorial units are autonomous entities and horizontally interconnected in bottom-up networks [16]. In contrast, China is a centralized state with a top-down administrative system, and the key feature of municipal amalgamations is a strong state presence [17–20]. Hence, China is a particularly interesting case to study, as it makes an important contribution to the literature on the interface between administrative restructuring and urbanization. Exploring the intrinsic motivations behind China’s administrative reorganization and examining the inner logic behind how China promotes urbanization through municipal amalgamations provides an in-depth understanding of the government-led urbanization development pattern there and a valuable reference for other developing countries seeking to formulate urbanization strategies.

Since implementing the reform and opening-up policy, China has established a reform of the city-county system to promote industrialization and urbanization. Since then, a variety of administrative reforms have taken place in China, including province-managing-county (PMC) reform, county-to-city upgrading reform, city-county mergers reform, etc. The policy effects of such quasi-natural experiments have been extensively explored by scholars. Bo examines the 1983 reform of the city-county system and its effect on regional development, demonstrating that the centralization of cities reduce regional resource misallocation and increase aggregate productivity [21]. Li et al. find that the reform of PMC has increased revenues for county governments, but the associated expansion of control power makes it difficult for upper-level governments to coordinate and supervise county governments, negatively affecting the economic performance [22]. Jia et al. analyze the creation of the Chongqing municipality in 1997 and suggest that increasing local government power by enhancing political hierarchy will lead to regional development [23].

A number of studies in China have provided useful references for understanding the effects of different types of municipal amalgamations. However, they mainly focus on the administrative reorganization in the early years of the reform and opening-up policy. During the past few years, city-county mergers have been the most common form of municipal amalgamations, and a growing number of researchers have explored how governments influence urban development through policy. To our knowledge, existing studies have mainly focused on "quantitative" aspects such as population agglomeration [24], economic growth [25–27], and housing prices [28], reflecting the "GDP-oriented" priorities of local governments, with insufficient attention to urban "quality" aspects. The most relevant paper for our study is Liang et al., who find that the city-county mergers in China are beneficial to basic education services, but have an unclear impact on health care services [29]. Despite being a valuable study, their research design is simple in that it lacks a comprehensive analysis of the transmission mechanism, and ignores the policy effect on the level of integrated public services. It is still necessary to develop a systematic and insightful analysis of how city-county mergers impact urban public services, as well as relevant theoretical and empirical studies.

As a complement to previous studies, our paper focuses on the "quality" of public services rather than quantifiable economic performance. The study is designed to answer what and how city-county mergers affect the quality of public services in China. The remainder of this paper is structured as follows. Section 2 is the background and theoretical hypotheses. Section 3 presents the research design, including the identification strategy, variables selection, and data descriptions. Section 4 presents the empirical results and analysis to validate the theoretical analysis. Section 5 is a case study in Ningbo City to illustrate the transmission mechanism between city-county mergers and the quality of public services. Section 6 contains the discussion and conclusion.

## Background and hypotheses

### Background

Since the implementation of the reform and opening-up policy, the urbanization rate in China has increased from 17.9% in 1978 to 64.72% in 2021, and the resident urban population has grown from 170 million to 914 million, representing a transfer of 744 million rural inhabitants to urban areas. With rapid economic and social development in rural areas, China has not only had one of the world’s most successful rural experiences, but it has also been successful in urbanizing its population. Especially since joining the WTO in 2001, China’s place in the global economy has become increasingly prominent.

China’s top-down, hierarchical urban management system provides an important institutional guarantee for urbanization’s rapid development and distinguishes its urban management from that of western countries. The adjustment of administrative boundaries in China is an important policy tool for resolving management conflicts, reducing administrative costs, and even promoting urbanization [24]. It has been argued that municipal mergers in developed western countries are motivated primarily by market integration effects and the expansion of market size. These factors are not relevant in China, where municipal governments implement municipal merger policies primarily out of three considerations: urban spatial development, financial interests, and political promotion [30].

Between 1980 and 1997, during the early years of the reform and opening-up policy in China, a series of administrative policy reforms were implemented to meet the urbanization development strategy of prioritizing small and medium-sized cities and small towns. This period was characterized by a county-to-city upgrading strategy that resulted in a substantial increase in the number of cities. Until 1997, the county-to-city upgrading policy was suspended due to pseudo-urbanization, massive agricultural land occupations, and suburban imbalances. City-county mergers were a smart strategy for meeting urban areas’ growing demand for production factors, and have been successfully adopted by local governments in recent years. Therefore, city-county mergers experienced a period of high tide between 2000 and 2002.

**Fig 1:**
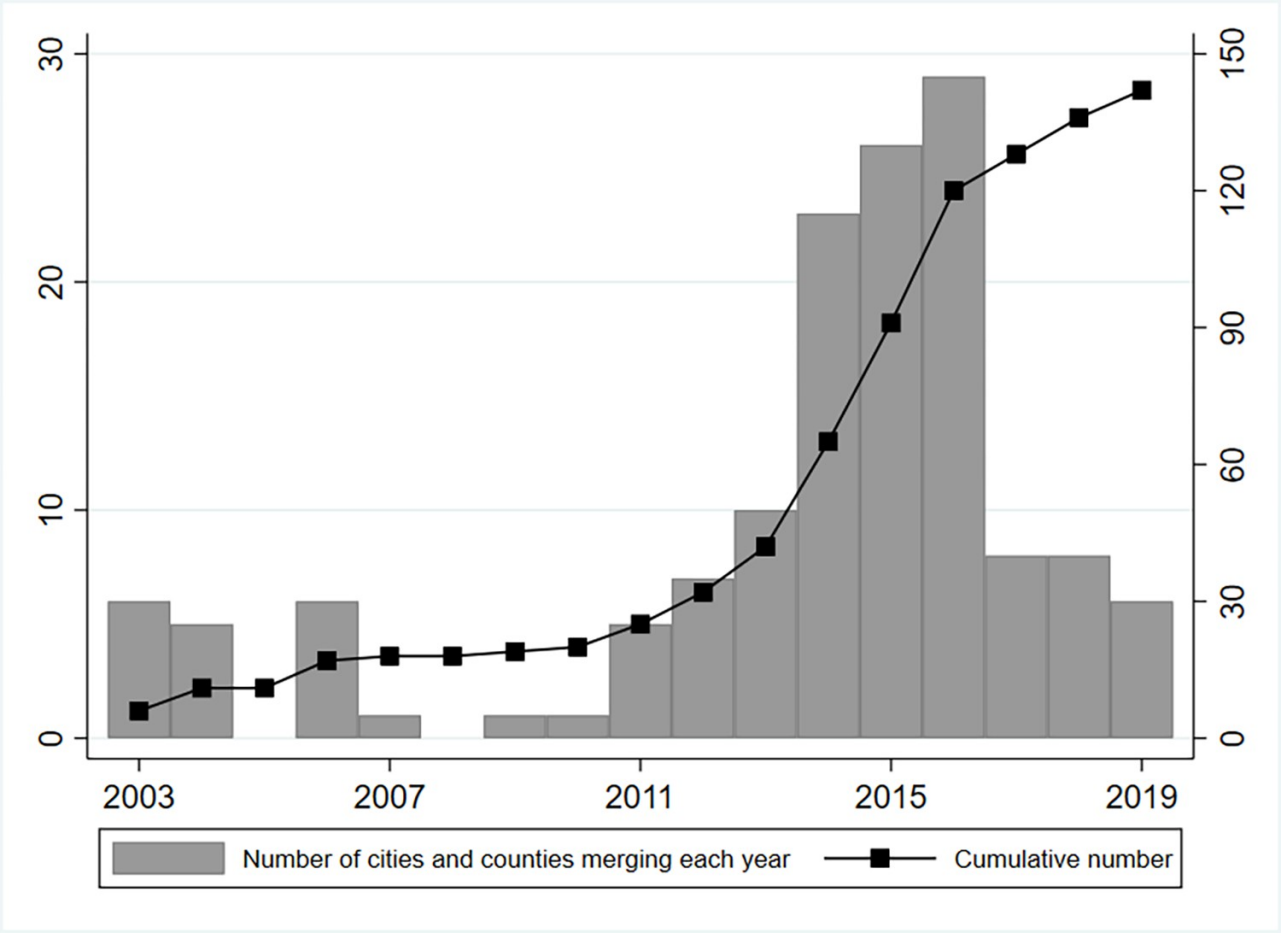
Number of cities with the policy of city-county mergers during 2003–2019.

**Fig 1** shows the change in the number of city-county mergers between 2003 and 2019. It can be seen that the practice of city-county mergers gradually peaked after 2012. It was because the Chinese Ministry of Finance proposed to implement the "strengthening counties and expanding authority" financial reform by the end of 2012, which would severely weaken the financial capacity of prefecture-level governments. A round of prefecture-level city-county mergers was initiated in the face of great financial pressure [32].

### Hypotheses

Under the top-down system, administrative power plays a greater role in resource allocation than market power does. According to the Chinese administrative hierarchy, cities are divided into four tiers: provincial, prefectural, county, and township. Cities with higher administrative hierarchies are more likely to receive resources. Embedded in the game of political promotion is the nature of economic competition, which has led to local protectionism and local market segmentation with administrative boundaries. Thus, administrative and economic regions display a high degree of congruence, while also exhibiting apparent conflict. The policy of city-county mergers has had an important impact on the quality of public services by changing the power structure and alleviating the inconsistency between administrative and economic areas. The paper analyzes the relationship between city-county mergers and public service quality from three perspectives.

First, the policy of city-county mergers has promoted an increase in the government’s finance capacity for public services by changing the allocation of financial power. The implementation of city-county mergers has broken the rigid administrative barriers between urban areas and their neighboring counties [24]. The financial power of the county-level government that is removed is transferred up to the municipal government, and the prefecture-level city government has unified decision-making power in urban planning, which enhances the leading role of local governments in optimizing the allocation of urban public resources. The merger of cities and counties followed by increased financial capacity from the municipal government has two positive effects on the quality of urban public services. In one sense, it guarantees the supply of funds for public services, and in another sense, it optimizes the efficiency of government financial expenditures in public services.

Second, the policy of city-county mergers has promoted an increase in livelihood expenditures and enhanced urban construction standards for public services by changing the configuration of administrative power. On one hand, since the policy of city-county mergers represent a small-scale decentralization process within the overall decentralization framework, it reduces the number of local governments in direct competition with one another [25]. The eliminated county governments are transformed from previously independent administrative units to livelihood-oriented service governments. The reduction in the autonomy of county governments has led to an increase in spending on people’s livelihoods, such as education, healthcare and municipal transport, which are closely linked to the welfare levels of residents [31]. On the other hand, the policy leads to an upgrade and gradual improvement in the functional positioning of the merged county. The level of public services is upgraded to municipal standards, with the construction of cultural centers, libraries, "triple-A" hospitals and transportation infrastructure receiving greater support from the national and municipal governments.

Third, the policy of city-county mergers has improved the demand for and supply of public services in cities by increasing the capacity for factor agglomeration. After the policy has been implemented, the total population of municipal districts shows "mechanism growth". The improvement in transportation infrastructure and in the economy of the urban agglomeration promotes the flow and effective distribution of amenities within the new administrative boundaries, leading to population clustering and further economic growth under the action of market mechanism [26]. A study finds that city-county mergers in eastern China have led to 11.04% urban expansion through resource integration, diffusion, and economic consolidation, compared to 16.17% and 10.20% in central and western China, respectively [32]. The increase in population implies an increase in the tax-paying capacity of urban residents, as well as a rise in the demand for public services both in terms of quantity and quality. Local governments will invest more financial expenditures in the construction of urban public services facilities and infrastructure. At the same time, to attract and retain talents, local governments are motivated to increase investment in education, health care, and transportation infrastructure to improve the quality of urban public services.

Based on the above analysis, the proposed research hypothesis is that the policy of city-county mergers improves the quality of public services in cities, leading to improvements in education and culture, medical and health care, and transportation infrastructure construction capacity, ultimately enhancing the overall quality of public services in cities.

## Research design

### Identification strategy

The policy of city-county mergers is an administrative adjustment tool, which can be viewed as a quasi-natural experiment aimed at meeting urban development imperatives. The Difference-in-Differences (DID) method has usually been used to assess whether a policy is working by measuring the difference between treatment and control groups in terms of the quality of urban services. Parallel trend assumption is a necessary precondition of DID to ensure that the differences between groups due to the policy itself are not due to preexisting trends. It is assumed that the treatment and control groups do not differ significantly in treatment effects before the implementation of the policy. Considering the large heterogeneity among cities across the country, it is a challenge to meet the DID parallel trend assumption, and one of the most widely used methods to reduce selection bias is Propensity Score Matching (PSM, first proposed by Rosenbaum and Rubin, 1983 [33]). Thus, the empirical process in our study is divided into two steps.

First, the PSM method is used to match the control group with the treatment group. Using a logit (or probit) model, PSM estimates the probabilities of treatment and searches for a city *i* in the control group that closely matches city *j* in the treatment group based on the individual city characteristic variables set *X*. The paper uses control variables as covariances representing individual characteristics to calculate a propensity score (P-score) based on a logit regression model, then uses a kernel matching method to match a similar control group with the treatment group.

Second, we use the DID model in Formula (1) to estimate the policy effects on the matched samples. Since city-county mergers were implemented in different years, the Staggered DID model should be used for the empirical analysis.


$${LQ}_{it}=\beta_{0}+\beta_{1}{DID}_{it}+\gamma {CTRL}_{it}+{\mu CITY}_{i}+\omega {YEAR}_{t}+\delta_{it}$$ (1)

Where *LQ*_*it*_ denotes the quality of public services in the city i during period t, *DID*_*it*_ is the dummy variable for the implementation of the policy and is the core explanatory variable, *CTRL*_*it*_ is a set of control variables, and *β*_0_ is the constant term. To control for individual fixed effects and time fixed effects, *CITY*_*i*_ and *YEAR*_*t*_ are included in the model, with corresponding coefficients of *μ* and *ω*. *δ*_*it*_ is a random disturbance term, and the standard errors of model coefficient estimates are clustered to the city level to mitigate the possible serial auto-correlation and heteroscedasticity problem. The estimations of the regression coefficients are achieved by the two-way fixed effects model based on STATA 15.1.

### Variables selection

#### (1) Explanation of explanatory variables and introduction to the entropy method

The explained variable represents the quality of public services. Based on the above theoretical analysis, this paper argues that the policy of city-county mergers results in the improvement of education and culture, medical and health care, and transportation infrastructure construction capacity and ultimately improving the quality of public services. Therefore, the quality of public services is measured comprehensively through three aspects: education and culture (edu), health care (hlth), and transportation (trsp). Indices for the quality of public services are shown in **Table 1**. The entropy method is used to make a comprehensive measurement of the quality of public services and to calculate the indices for the three aspects separately.

**Table 1:** Quality of public services evaluation indices.

Category	Indicator	Indicator Code
Education and culture	Number of full-time teachers in elementary school (persons)	*X_1_*
	Number of full-time teachers in general secondary schools (persons)	*X_2_*
	Number of books in public libraries (volumes)	*X_3_*
Health care	Total number of licensed physicians and licensed assistant physicians (persons)	*X_4_*
	Number of hospital and health center beds (sheets)	*X_5_*
	Number of hospitals and health centers (pieces)	*X_6_*
Transportation	Actual road area at the end of the year (million square meters)	*X_7_*
	Total annual public bus (electric) passenger transportation (persons)	*X_8_*

Note: Indicators were selected by the authors based on relevance and availability of data.

The entropy method is an objective weighting method that assigns weights to different indicators based on the amount of information and degree of correlation in the original data. In determining the weighting coefficients, the method effectively avoids the bias caused by subjective factors to accurately show the importance of different indicators. An index with a lower entropy value provides more information and greater variation. Accordingly, the larger the weight, the greater the role, and vice versa [34]. The main steps in calculating the weight of an indicator based on the entropy method are as follows:

①Standardization of data. To eliminate the influence of magnitude and order of magnitude on the evaluation results, the data are first standardized using the extreme difference method. The positive indicator calculation method is used when a larger value of an economic indicator indicates higher levels of urban public services. The negative indicator calculation method is used when a larger value of an economic indicator indicates lower levels of urban public services. Considering that all eight tertiary indicators in this paper are positive, the standardization formula is as follows:

$$X_{ijk}\prime =\frac{X_{ijk}-{min\;(X}_{j})}{{max\;(X}_{j})-{min\;(X}_{j})}$$ (2)

Where *X*_*ijk*_ is the original value of the *j*th index in the *k*th city of the *i*th year, and *X*_*ijk*_′ is the standardized value (i = 1,2,…,m; j = 1,2,…,p; k = 1,2,…,n). *min* (*X*_*j*_) and *max* (*X*_*j*_) are the minimum and maximum values of the *j*th indicator for all cities in all years, respectively. The standardized data are shifted by 0.0001 to eliminate the effect of zero values.

②We calculate the share of indicator *w*_*ijk*_

$$\varpi_{ijk}=\frac{X_{ijk}\prime }{\sum_{k=1}^{n}\sum_{i=1}^{m}X_{ijk}\prime }$$ (3)

③We calculate the information entropy and information entropy redundancy of the *j*th indicator, *e*_*j*_ (0≤*e*_*j*_≤1) and *d*_*j*_

$$e_{j}=-\frac{1}{lnm}\sum_{k=1}^{n}\sum_{i=1}^{m}\left(\varpi_{ijk}\times ln\varpi_{ijk}\right)$$ (4)

$$d_{j}=1-e_{j}$$ (5)

④We calculate the weight of indicator *j*, *w*_*j*_

$$w_{j}=\frac{d_{j}}{\sum_{j=1}^{p}d_{j}}$$ (6)

⑤We calculate the composite indicator, *S*_*ik*_

$$S_{ik}=\sum_{j=1}^{p}{(w}_{j}X_{ijk}\prime )$$ (7)

#### (2) Explanatory and control variable descriptions

The core explanatory variable is *DID*_*it*_, which is a dummy variable for the implementation of the policy. *DID*_*it*_ = *treat*_*i*_×*post*_*t*_. *treat*_*i*_ = 1 if city i implemented the policy in the sample period and 0 otherwise; *post*_*t*_ = 1 if city i implemented the policy in year t and 0 otherwise.

To reduce the bias caused by the omission of variables, the following control variables are selected, accounting for the factors influencing the quality of public services that have been described in the literature:

Industrial structure (secondary industry and tertiary industry). Urban industrial development affects the level of urban development, which impacts public services in turn. The shares of secondary and tertiary industries’ output value in GDP are used as proxy variables for industrial structure in this paper.

City scale. The size of the urban population reflects residents’ demand for public goods. More people who pay taxes for public services means more urban public services can be provided. The city scale is expressed using the logarithm of the registered population.

Human capital. Human capital represents the knowledge that is embodied in labor, such as knowledge, cultural and technical skills, health status, etc. By enhancing their capacity and skills, workers can increase productivity, which contributes to urban economic development and provides more tax revenue for public services. Considering the availability of data, we use the total number of education years of local elementary school students (pri) and junior high school students (sec) as the measure for the level of human capital, which is calculated as *hum* = ln (pri*6+sec*9).

GDP per capita. Per capita income is a measure of the standard of living among city dwellers. People with a high per capita income will tend to live in a city with a high standard of living, as well as expect a high standard of public services. GDP per capita is expressed using the logarithm of per capita income.

Financial level. Through the facilitation of asset transactions, the reduction of financing costs and liquidity risks, and the enhancement of resource allocation efficiency, a well-developed financial system can assist urban development and improve public services. The level of financial development is represented by the ratio of financial institutions’ loan balances to regional GDP.

Government revenue. The financial capacity of a government directly affects the ability of a city to provide public services. The more financially strong a city government, the more money it can spend on the construction of urban municipal facilities, promoting an improved quality of public services. Government revenue is expressed using the logarithm of local government general budget revenue.

### Data descriptions

An empirical analysis of city-county mergers was conducted using unbalanced panel data from 278 cities at the municipal district level from 2003 to 2019. The year 2003 was chosen because county-city upgrading and prefecture-level city abolition almost ceased after 2003. China’s prefecture-level city population had become stable since then. By the end of 2019, there were 297 prefecture-level cities in China (including Beijing, Shanghai, Tianjin, and Chongqing, which are directly under the authority of the central government). Based on data availability and completeness, 19 cities were excluded, resulting in unbalanced panel data for 17 years and a sample of 278 individuals. It excluded Sansha, Danzhou, Bijie, Tongren, Haidong, Turpan, Hami, Zhongwei, Lincang, Urumqi, Karamay, Hulunbeier, Longnan (13), and all six prefecture-level cities in the Tibet Autonomous Region (6). By using the Propensity Score Matching Difference-in-Differences model (PSM-DID), the paper identified the causal relationship.

The list of cities implementing the policy and the years during which it was implemented was compiled from China’s administrative division website (http://www.xzqh.org) and the Chinese central government website (www.gov.cn). The actual study identified 95 cities that implemented the policy between 2003 and 2019, after excluding cities for which data were missing.

Most of the data were obtained from the EPS China Urban Database. Using 2003 as the base period, we deflated the variables measured in currency using the GDP deflator for the province where the city is located. To reduce heteroskedasticity and dispersion of the sample data, we used the logarithm of the variables for non-percentage measures, including variables for city scale, human capital, GDP per capita, and government revenue. The descriptive statistics of variables are shown in Table 2.

**Table 2:** Descriptive statistics of variables.

Variable	Unit	N	Mean	Standard deviation	Min	Max
Public services	None	4375	0.0517	0.0696	0.0044	0.7140
Education and culture	None	4375	0.0200	0.0266	0.0003	0.2840
Health care	None	4375	0.0184	0.0248	0.0012	0.3000
Transportation	None	4375	0.0134	0.0206	0.0002	0.2100
Secondary industry	%	4375	0.4940	0.1200	0.0857	0.9040
Tertiary industry	%	4375	0.4350	0.1120	0.0861	0.8350
City scale	Million people	4357	1.3480	1.5450	0.0010	14.620
Human capital	None	4375	2.0030	0.3300	2.7900	27.290
GDP per capita	Million Yuan	4375	0.0483	0.0368	0.0025	0.4680
Financial level	%	4375	1.1820	0.6390	0.0428	8.8940
Government revenue	Billion Yuan	4375	10.650	38.060	0.0269	710.80

## Results analysis

### Sample matching

Based on Heyman et.al [35]and Zhang et.al [36], we use a year-by-year matching approach to match the treatment group to the control group for each year and exclude control samples that did not match. Based on the logit regression model, we obtain the P-score (propensity score) of the control variables; using the kernel matching method, we match the treatment and control groups.

To verify the reliability of the matching results, a balance test is conducted on the matching results for each year. The results for 2003 are presented in **Table 3.** According to the T-test results, most covariates differ significantly between the treatment and control groups before matching, indicating that the PSM method is required. While after PSM based on the kernel matching method, the covariates become balanced between two groups, with deviations of all variables within 10% and p values significantly greater than 1%. Thus, the treatment and control groups are comparable after PSM, and the balance assumption is met.

**Table 3:** PSM balance test results in 2003.

Variable	Unmatched Matched	Mean		%bias	T-test	
		Treated	Control		t	p>|t|
Secondary industry	U	0.523	0.493	25.3	1.94	0.053
	M	0.526	0.522	2.80	0.20	0.845
Tertiary industry	U	0.411	0.403	8.10	0.64	0.522
	M	0.408	0.411	-3.20	-0.21	0.834
City scale	U	4.636	4.344	38.7	3.13	0.002
	M	4.567	4.507	7.90	0.54	0.588
Human capital	U	2.046	1.969	22.5	1.82	0.070
	M	2.023	2.010	3.9	0.26	0.796
GDP per capita	U	9.807	9.411	63.4	4.92	0.000
	M	9.786	9.737	7.90	0.57	0.568
Financial level	U	1.401	1.170	40.5	3.22	0.001
	M	1.339	1.415	-13.3	-0.85	0.394
Government revenue	U	11.526	10.870	48.6	3.98	0.000
	M	11.416	11.331	6.30	0.42	0.672

Note: T values adjusted for clustered heteroskedasticity are in parentheses.

**Fig 2:**
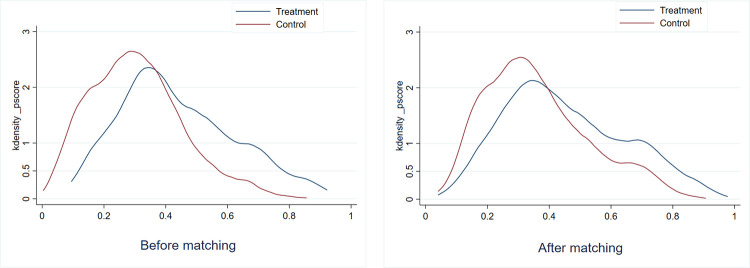
Comparison of kernel density distributions of P-score.

To demonstrate visually the matching results between the treatment group and the control group before and after propensity score matching, the kernel density distributions of the P-score are shown in **Fig 2**. Consequently, the distribution similarity between the treatment group and the control group is higher after PSM, indicating reduced sample selection bias.

### Benchmark regressions

Following PSM matching of treatment and control groups, DID regression analysis is performed on the matched samples. **Table 4** shows the results. PSM-DID regression results are shown in columns (1) and (2), with the second column adding control variables. As control variables are added, the model’s explanatory strength increases from 0.437 to 0.484, and the coefficients of policy DID are all positive at the 1% significance level. Column (2) shows the results of the baseline regression, with a coefficient of 0.01 on the DID, implying that the city-county mergers boost the index of urban public services by an average of 1% units.

**Table 4:** Evaluation results of the policy impact on the quality of public services (Explained variable: Public services).

Model	(1)	(2)	(3)	(4)
	PSM-DID	PSM-DID	DID	DID
DID	0.019***	0.010***	0.024***	0.015***
	(7.11)	(2.76)	(7.24)	(3.84)
Secondary industry		-0.016		-0.017
		(-0.88)		(-1.06)
Tertiary industry		-0.016		-0.008
		(-0.79)		(-0.44)
City scale		0.004		0.005
		(1.35)		(1.38)
Human capital		0.039***		0.037***
		(5.53)		(5.12)
GDP per capita		-0.001		0.000
		(-0.55)		(0.13)
Financial level		0.002*		-0.000
		(1.86)		(-0.01)
Government revenue		-0.002		-0.004**
		(-1.19)		(-2.03)
_cons	0.033***	-0.016	0.036***	-0.005
	(31.21)	(-0.64)	(30.45)	(-0.17)
City fixed effects		YES		
Year fixed effects		YES		
N	4022	4022	4375	4357
R^2^	0.437	0.484	0.404	0.442

Note: T values adjusted for clustering heteroskedasticity are in parentheses, and ***, **, and * indicate significance at the 1%, 5%, and 10% levels, respectively.

As a comparison, Columns (3) and (4) present the regression results for the DID model, with the last column adding control variables. The regression results further confirm the robustness of the study findings, indicating that city-county mergers have a positive impact on urban public services.

### Parallel trends and dynamic effects tests

As a prerequisite to applying the Staggered DID method, the treatment and control groups must satisfy the parallel trend hypothesis. This means there are no significant differences in the level of urban public services between the treatment and control groups when no external shock is present from city-county merger policies. A parallel trends test is conducted according to the event analysis approach developed by Beck et al. [37].


$${PS}_{it}=\gamma_{0}+\sum_{1}^{15}\gamma_{j}{pre}_{j}+\rho_{0}current+\sum_{1}^{16}\rho_{k}{post}_{k}+\sum {CTRL}_{it}+{\mu CITY}_{i}+\omega {YEAR}_{t}+\delta_{it}$$ (8)

Where *pre*_*j*_, *current* and *post*_*k*_ are time dummy variables relative to the year of policy implementation. *pre*_*j*_ = 1 means the current year of the city is the *j*th year before the policy implementation, otherwise, it takes the value of 0. *current* = 1 means the current year of the city is the first year of the policy implementation, otherwise, it means the current year is not the first year of the policy implementation. *post*_*k*_ = 1 means the current year of the city is the *k*th year after the policy implementation, otherwise it takes the value of 0. The rest of the variables are consistent with the model (1). To avoid multicollinearity and falling into the dummy variable trap, the first year before policy implementation (*pre*_1_) is chosen as the base period.

**Fig 3:**
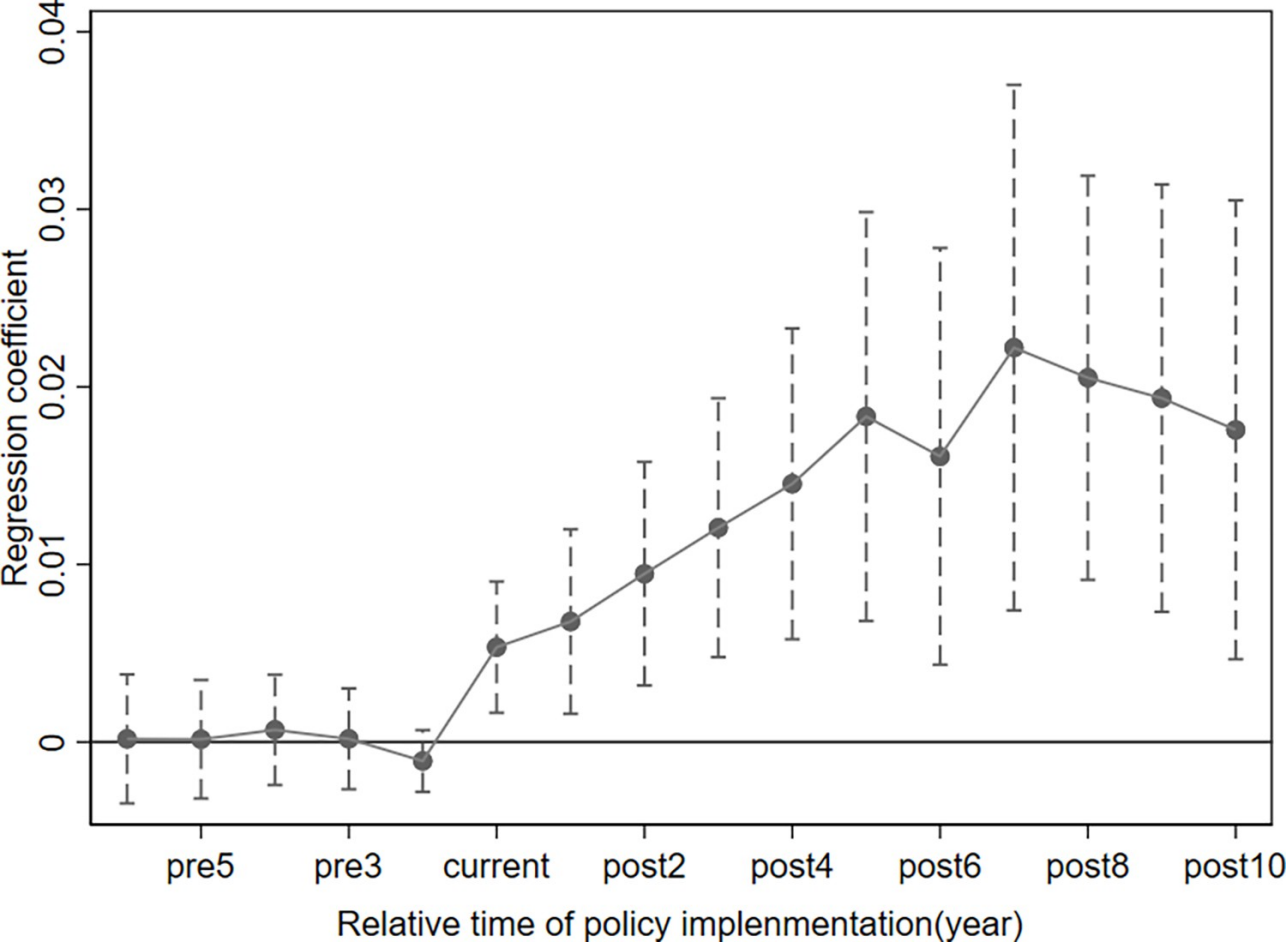
Results of the parallel trends and dynamic effects tests.

A visualization of the results is presented in **Fig 3** which depicts the dynamic effects of the city-county mergers policy in different years. As can be seen, the 95% confidence interval for the regression coefficients before implementation of the policy covers a value of 0, while the coefficients after the implementation are significantly positive. The results indicate that there is no significant difference between the treatment and control groups after PSM before the policy implementation, supporting the hypothesis of parallel trends. After the policy has been implemented, policy effects begin to emerge, confirming that the policy has improved public services.

As a result of the dynamic effects of the policy, over the five years following the implementation of the policy, the impact of city-county mergers on the quality of public services in urban areas gradually increases. After the fifth year, the policy effect tends to decline and gradually stabilize. This dynamic effects could be caused by the government’s re-planning and coordination of public resource allocation, which requires detailed internal validation and approval processes, resulting in a time lag. In the first five years, it increased year on year. Afterward, the effect of the policy levels off in urban areas as public services improve.

### Robustness checks

#### (1) Different approaches to matching

To ensure the robustness of the study findings, three propensity score matching methods are used to match the control and treatment groups, including first-order nearest neighbor matching, caliper matching, and Mahalanobis matching. The results of the PSM-DID model are shown in **Table 5**. Based on the estimation results, the coefficients estimated for the main variables under different matching methods are consistent with the baseline regression estimates. Therefore, it can be concluded that the significant positive impact of city-county mergers on the urban quality of public services is robust.

**Table 5:** Robustness of test results based on different approaches to matching (Explained variable: Public services).

Model Matching approaches	(1)	(2)	(3)
	PSM-DID	PSM-DID	PSM-DID
	Fourth order nearest neighbor matching	Radius matching	Mahalanobis matching
DID	0.008**	0.006**	0.007*
	(2.04)	(2.02)	(1.81)
Secondary industry	-0.012	-0.020	-0.015
	(-0.59)	(-1.18)	(-0.61)
Tertiary industry	-0.013	-0.022	-0.018
	(-0.57)	(-1.15)	(-0.66)
City scale	0.003	0.004	0.003
	(1.05)	(1.52)	(1.03)
Human capital	0.040***	0.036***	0.044***
	(5.04)	(5.52)	(5.16)
GDP per capita	-0.001	0.000	-0.002
	(-0.31)	(0.02)	(-0.68)
Financial level	0.002**	0.002**	0.003**
	(2.00)	(2.05)	(2.07)
Government revenue	-0.003	-0.002	-0.004
	(-1.36)	(-0.98)	(-1.59)
_cons	-0.011	-0.023	0.001
	(-0.37)	(-0.92)	(0.03)
City fixed effects	YES		
Year fixed effects	YES		
N	3453	3650	3464
R^2^	0.484	0.475	0.501

Note: Radius matching is set at a radius of 0.01. T values adjusted for clustering heteroskedasticity are in parentheses, and ***, **, and * indicate significance at the 1%, 5%, and 10% levels, respectively. M = 4 for Mahalanobis matching.

#### (2) Placebo test

Despite controlling for many city characteristic variables in the benchmark regressions, there may still be unobserved variables or random factors that interfere with study findings and therefore invalidate the identification hypothesis. A placebo test is adopted as an indirect test to ensure the robustness of the study findings. The placebo test relies on the assumption that the regression coefficients of placebo treatment variables do not deviate significantly from zero if there is no significant omitted variable bias. A dummy variable for the placebo test is constructed by generating a randomized policy implementation time for the randomly selected treatment group, and this randomization process is repeated 500 times to improve identifiability.

**Fig 4:**
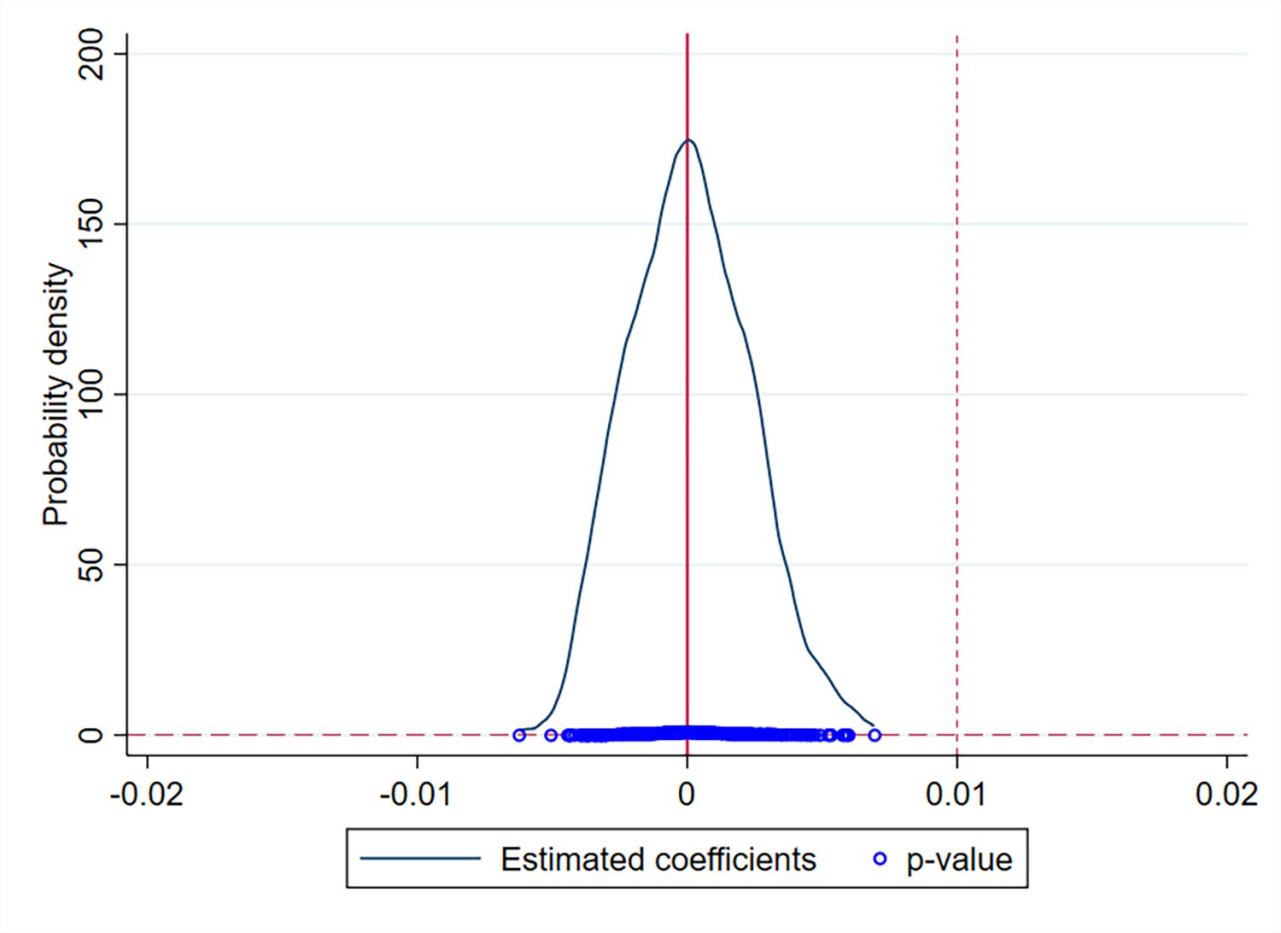
Distributions of estimated coefficients and p values based on placebo test.

**Fig 4** shows the estimated kernel densities of the coefficients and the corresponding p values distributions for the 500 randomly generated treatment groups. The regression coefficients are clustered around the value of 0, and most p values are greater than 0.1. The dashed line in **Fig 4** represents the estimated coefficient of 0.01 in the baseline regression, which is an outlier compared to that of the placebo test.

The placebo test implies that the positive effects of the policy on the urban quality of public services are not influenced by other unobserved variables or random factors. Consequently, the findings are confirmed as robust.

### Further tests

#### (1) A test of different public services

To further analyze the heterogeneity of the policy effects on the quality of public services, we divide public services into three aspects, education and culture, health care, and transportation, using the PSM-DID model to estimate. The regression results are shown in Table 5. For education, health care, and transportation, the regression coefficients are 0.004, 0.003, and 0.003, respectively, and they are significant at 1%.

The Seemingly Unrelated Regression (SUR) tests for differences in coefficients between groups, and its results are presented in the last row of **Table 6**. It can be seen that the positive effects of the city-county mergers on education and culture are significantly stronger than that on transportation. There is no significant difference between health care and education, or health care and transportation when city-county mergers occur.

**Table 6:** A comparison of the policy impact on different aspects of public services.

Model Explained variable	(1)	(2)	(3)
	PSM-DID	PSM-DID	PSM-DID
	Education and culture	Health care	Transportation
DID	0.004***	0.003**	0.003**
	(2.81)	(2.16)	(2.27)
Secondary industry	-0.003	-0.008	-0.006
	(-0.47)	(-0.79)	(-0.90)
Tertiary industry	-0.004	-0.010	-0.003
	(-0.57)	(-0.90)	(-0.43)
City scale	0.002	0.003**	-0.001
	(1.45)	(2.15)	(-0.61)
Human capital	0.024***	0.010***	0.006***
	(6.58)	(3.96)	(2.76)
GDP per capita	-0.001	0.000	-0.000
	(-1.57)	(0.49)	(-0.27)
Financial level	0.000	0.001*	0.001
	(0.68)	(1.82)	(1.51)
Government revenue	-0.001*	-0.001	-0.000
	(-1.74)	(-1.07)	(-0.16)
_cons	-0.013	-0.007	0.003
	(-1.38)	(-0.53)	(0.40)
City fixed effects			
Year fixed effects			
N	4022	4022	4022
R^2^	0.449	0.311	0.386
SUR test	Edu. versus Health. 2.32	Edu. versus Trans. 5.66**	Health. versus Trans. 0.59

Note: T values adjusted for clustering heteroskedasticity are in parentheses, and ***, **, and * indicate significance at the 1%, 5%, and 10% levels, respectively.

#### (2) A test of public services per capita

To further test the effect of city-county mergers on the quality of public services per capita, we divide each indicator in **Table 1** by the resident population and recalculate the explanatory variables using the entropy method. Based on the EPS China Urban Database, the data on resident population is calculated using the GDP divided by GDP per capita ratio for municipal districts.

PSM is conducted using the kernel density method in line with the benchmark regressions in **Table 4**. The estimation results based on PSM-DID and DID model are shown in **Table 7**. Based on the results shown in the table, the coefficients on DID are significantly negative for both the PSM-DID and DID methods. PSM-DID with control variables in column (2) is the main regression, with a coefficient of 0.004 for DID, implying that city-county mergers result in a decline of 0.4% in public services per capita. It is less than 1% for the overall public services. However, it does not mean that city-county mergers negatively affect public services. It is possible that the increased public resources brought about by city-county mergers have led to a greater concentration of population, indicating a greater intensity of population concentration than of public resources.

**Table 7:** Evaluation results of the policy impact on the quality of public services per capita (Explained variable: Public services per capita).

Model	(1)	(2)	(3)	(4)
	PSM-DID	PSM-DID	DID	DID
DID	-0.009***	-0.004***	-0.009***	-0.005***
	(-6.79)	(-3.32)	(-7.35)	(-4.08)
Secondary industry		-0.171***		-0.154***
		(-2.75)		(-3.17)
Tertiary industry		-0.131**		-0.116**
		(-2.16)		(-2.54)
City scale		-0.002		-0.001
		(-0.76)		(-0.50)
Human capital		0.005		0.007*
		(1.42)		(1.88)
GDP per capita		0.053***		0.051***
		(4.16)		(4.32)
Financial level		0.007***		0.007***
		(3.24)		(3.66)
Government revenue		-0.006***		-0.007***
		(-2.98)		(-3.17)
_cons	0.048***	-0.260***	0.048***	-0.257***
	(83.10)	(-4.73)	(84.76)	(-4.41)
City fixed effects			YES		
Year fixed effects			YES		
N	4022	4022	4357	4357
R^2^	0.087	0.336	0.094	0.332

Note: T values adjusted for clustering heteroskedasticity are in parentheses, and ***, **, and * indicate significance at the 1%, 5%, and 10% levels, respectively.

## Analysis of the transmission mechanism

The above theoretical and empirical analyses confirm that city-county mergers significantly contribute to the improvement of the urban quality of public services. To further sort out the inner transmission mechanism, we have conducted a qualitative analysis based on the case of Fenghua District in Ningbo City. Financial data for Ningbo are from the Ningbo Statistical Yearbook published by the Ningbo Bureau of Statistics (http://tjj.ningbo.gov.cn/col/col1229042824/index.html), and financial data for Fenghua District are from financial statistics published by the Fenghua District Bureau of Statistics (http://www.fh.gov.cn/col/col1229045208/index.html).

**Fig 5:**
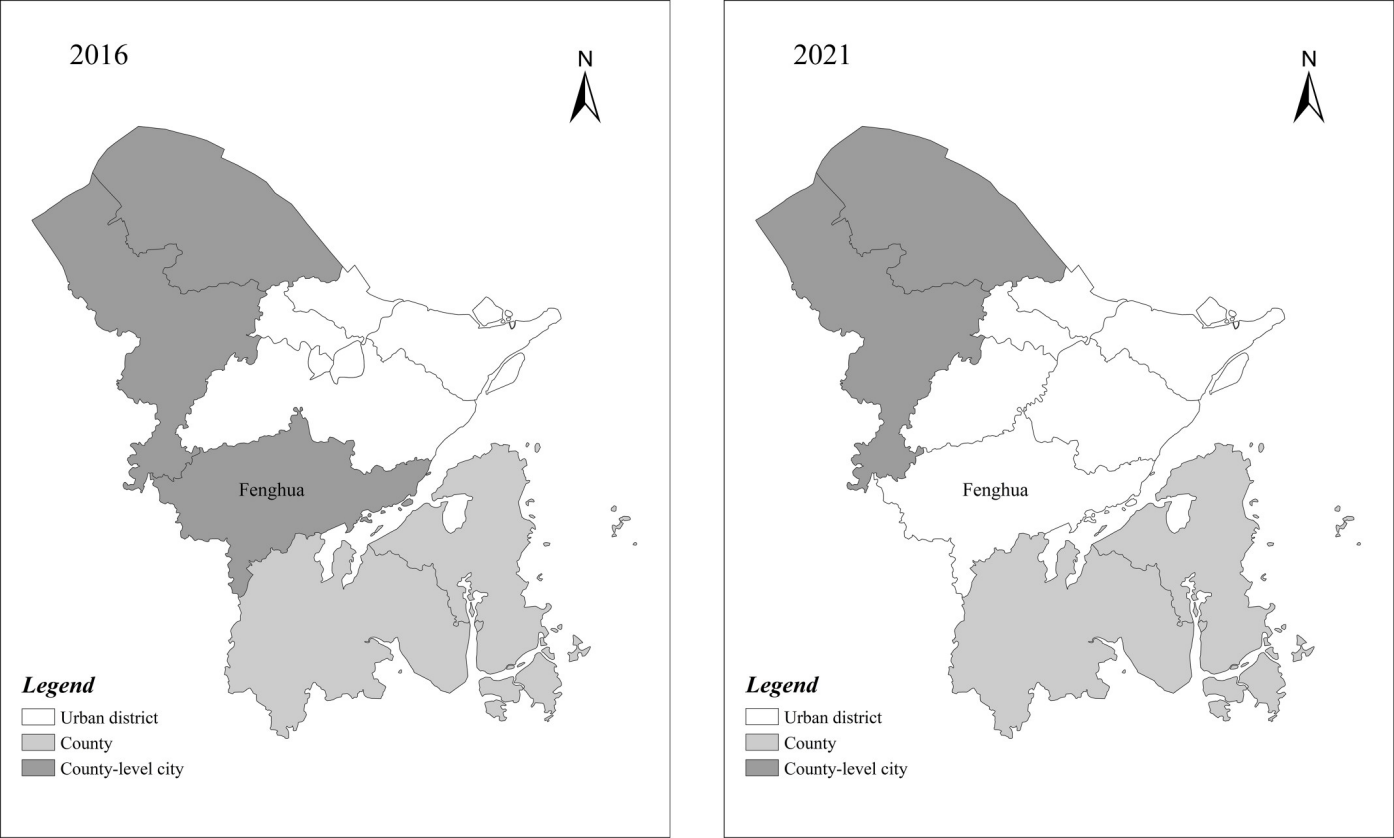
Comparison of administrative divisions in Ningbo, Zhejiang Province, 2016 and 2021.

In September 2016, the State Council of China approved the adjustment of Ningbo’s administrative division, abolishing Fenghua County and establishing the Ningbo Fenghua District, the last district to be established in Ningbo. The Fenghua District is located in the eastern portion of Zhejiang Province and is the southern suburb of Ningbo City, comprising 30.3% of the area under Ningbo’s jurisdiction. It is the largest municipality in Ningbo and today consists of eight streets and four towns. By implementing the city-county merger policy, Fenghua becomes a municipal district from a county-level city, and the area of Ningbo city grows by 1277km^2^ (the area of Fenghua district). **Fig 5** shows the changes in the administrative division of Ningbo City in 2006 and 2021 after the implementation of the city-county merger in the Fenghua District.

As a result of the city-county merger policy, the original structure of financial and administrative power between Ningbo and the former Fenghua County has changed. The quality of public services is affected by the policy from the following two perspectives.

**Fig 6:**
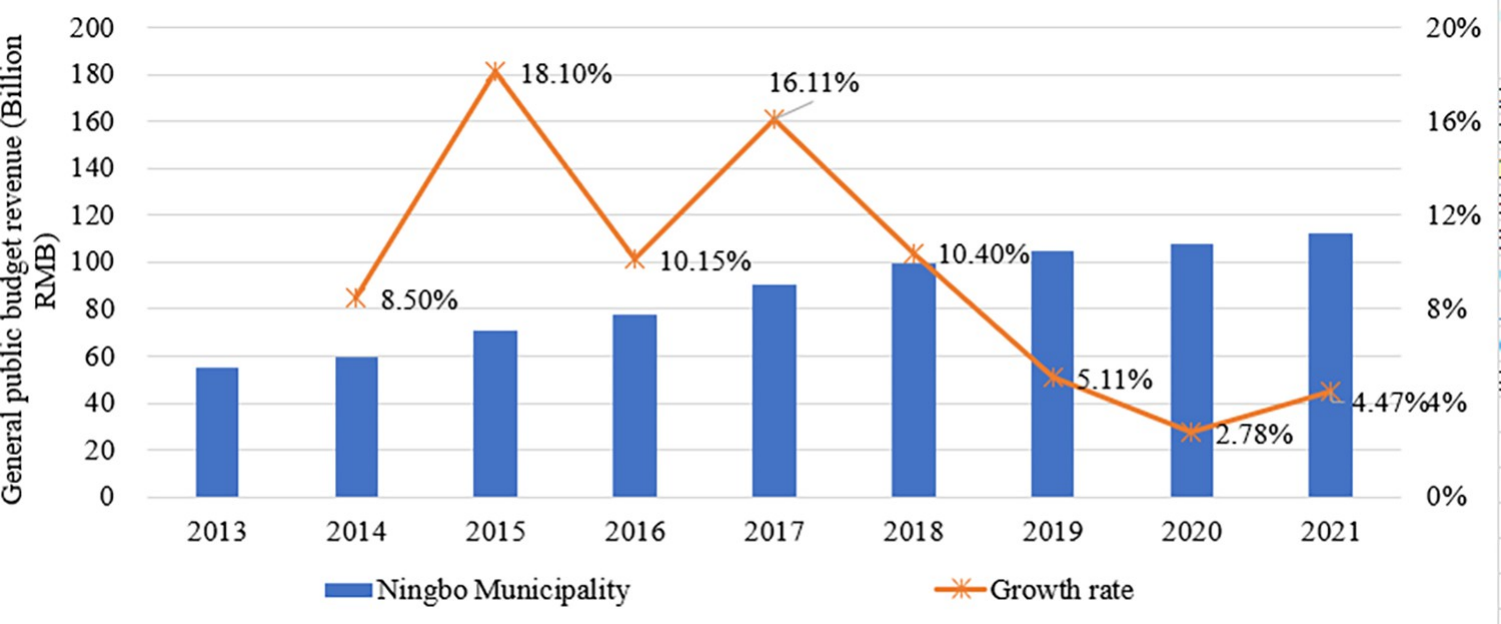
Changes in general public budget revenue and growth rate in Ningbo municipality, 2013–2021.

(1) One channel by which the city-county mergers policy has affected public services is through the allocation of financial power. The city-county merger policy has increased the financial power of the Ningbo municipal government and provided financial support for public services quality improvement. **Fig 6** shows the changes in public finance budget revenues and growth rate of the Ningbo municipal government for the four years before and after the implementation of the policy (2013–2021), with 2017 as the middle year (the first year of policy implementation). It can be seen that public budget expenditure shows a jump in 2017, with the growth rate increasing from 10.15% to 16.11%. The graph also shows a jump in 2015, which is most likely caused by the addition of two new towns to the Beicang District in October 2014. After 2017, the growth rate gradually slows down and stabilizes, maintaining a lower growth rate in 2020 and 2021 mainly due to the impact of COVID-19.

To further analyze the sources of the changes in Ningbo in 2017, **Table 8** gives a comparison of public budget revenue by the district in 2016 and 2017. There are two 0 values in the table. One is the 0 value for Fenghua District in 2016, which was due to Fenghua District still being a county-level administrative unit at that time; another 0 value is for Jiangdong District in 2017, because it was abolished and merged to Haishu District and Yinzhou District in 2016. The data in the table shows that the increase in public budget revenue in the urban area of Ningbo is 12.521 billion RMB, of which the total increase in the municipal districts accounted for 80.28%. The increase in fiscal revenue from Haishu District and Yinzhou District is roughly offset by the decrease from the abolished Jiangdong District. 34.29% of the increase in fiscal revenue is contributed by Fenghua District, with the other three districts have also made a great contribution. Nevertheless, it can be argued that the jump in financial revenue growth in Ningbo in 2017 is directly and closely related to the merging of the Fenghua District.

**Table 8:** Comparison of public budget revenue of Ningbo Municipality, 2016 and 2017.

Public budget revenue (Billion RMB)	2016	2017	Increments	Incremental share
Fenghua	0	4.294	4.294	34.29%
Haishu	5.165	9.302	4.138	33.05%
Jiangdong	6.766	0	-6.766	-54.04%
Jiangbei	6.001	6.502	0.501	4.00%
Beilun	20.736	24.659	3.923	31.33%
Zhenhai	6.471	7.048	0.576	4.60%
Yinzhou	20.778	24.165	3.387	27.05%
Municipal districts	65.917	75.97	10.053	80.28%
Urban area	77.737	90.258	12.521	100%

Note: The incremental share is the proportion of incremental growth in each district compared to the incremental growth in the urban area.

**Fig 7:**
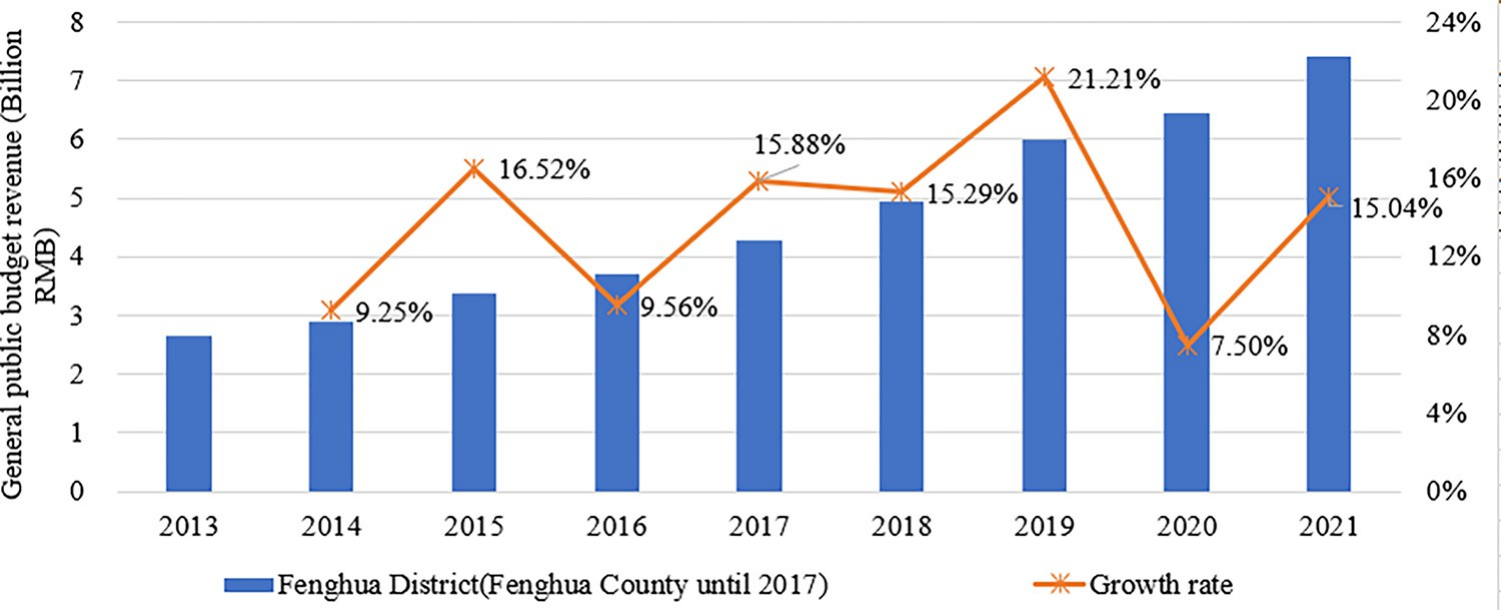
Changes in general public budget revenue and growth rate in Fenghua District (County), 2013–2021.

**Fig 7** compares and analyzes the changes in general public budget revenue and growth rate in Fenghua District (Fenghua County until 2017) from 2013 to 2021. The general public budget revenue has increased steadily over the past few years. Following the implementation of the city-county merger policy in 2016, the growth rate increased from 15.88% to a maximum value of 21.21% in the first three years. It indicates that the policy has improved the financial power of Fenghua District (County) to some extent, by which the government can provide better financial support for improving the quality of public services.

**Fig 8:**
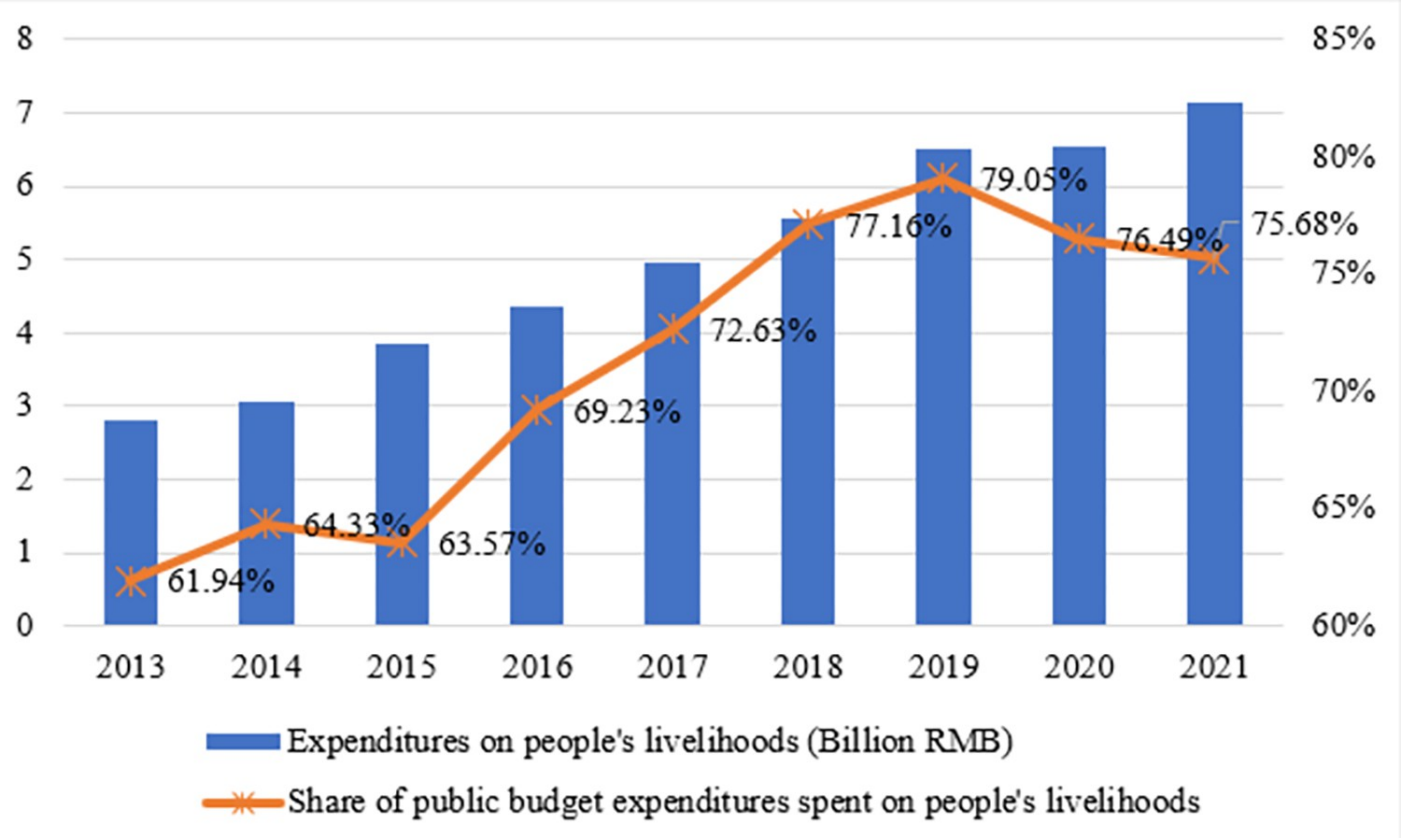
Changes in the expenditures on people’s livelihood and the share in public budget expenditures in Fenghua District (County), 2013–2021.

(2) Another channel by which the city-county mergers policy has affected public services is through the allocation of administrative power. The policy has transformed Fenghua County from a relatively independent county-level administration into a district-level administration serving the City of Ningbo, allowing the government to focus more on improving public services and residents’ welfare than on economic development. **Fig 8** illustrates the amount and percentage changes of public budget expenditures on people’s livelihood in Fenghua District (County) between 2013 and 2021. It can be seen that since 2013, the livelihood expenditures and the proportion of public budget expenditures in Fenghua District (County) have shown steady growth. However, following the implementation of the policy in 2016, that share increases to 69.23%. In particular, over the first three years after the implementation of the city-county merger, the shares have increased by as much as 72.73% 77.16%, and 79.05% respectively, indicating the correlation between the implementation of the city-county merger and the growth of the livelihood expenditures in Fenghua District.

**Fig 9:**
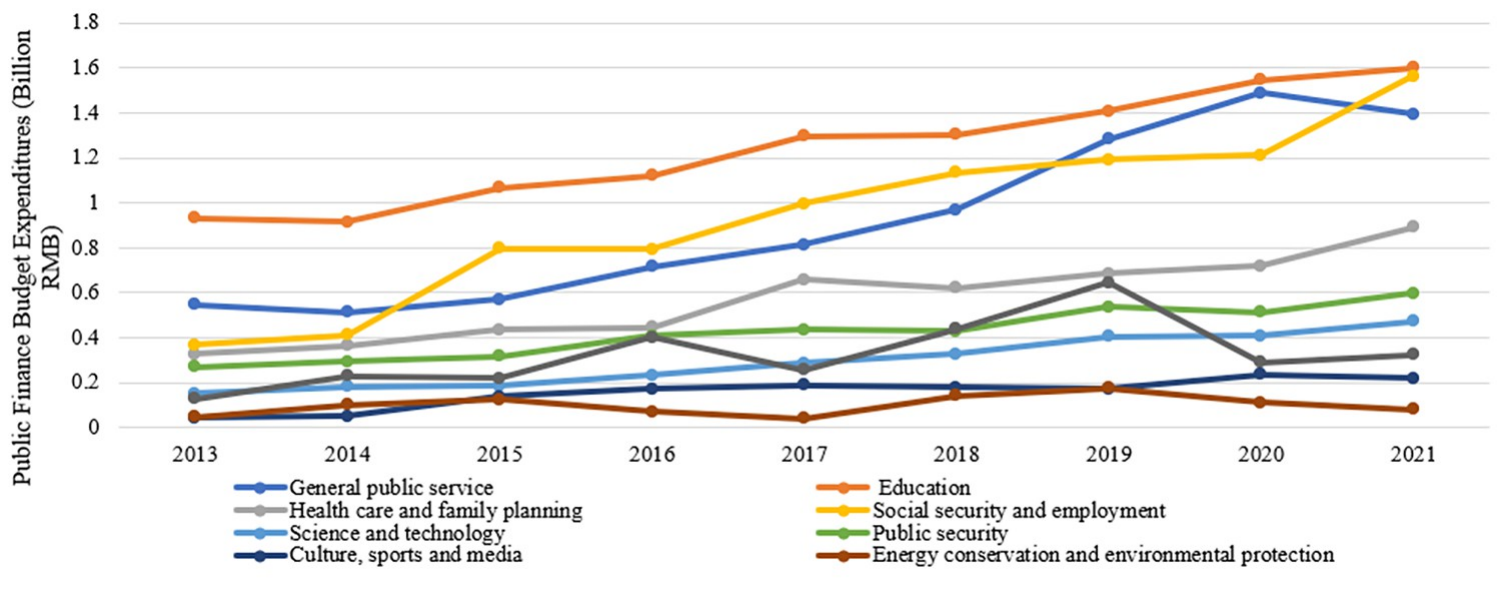
Comparison of the nine livelihood expenditure items in Fenghua District (County), 2013–2021.

Expenditures on people’s livelihood mostly include the nine items in **Fig 9**. The rest of the public budget expenditures are non-livelihood expenditures (productive expenditures), which mainly stem from transportation, agriculture, forestry and water issues, and commercial services affairs. As can be seen, the growth in livelihood expenditures since 2017 is steeper than in the previous periods. In these categories, education is the biggest expenditure category and has been increasing year after year. After 2017, expenditures on education, social security and employment, general public service, and health care all increased significantly. It shows that the city-county merger has resulted in increased expenditures on education, healthcare, and other livelihood categories in the Fenghua District.

Furthermore, the change in the allocation of administrative power in Fenghua District has reflected in the upgraded city’s functional positioning. Since the district was merged into Ningbo City, urban planning and construction in Fenghua District are now under the jurisdiction of the Ningbo municipal government rather than the former county government. The overall development plan for Fenghua District in 2018 elevated it to "the central city of southern Ningbo", reflecting its integration within Ningbo central city. The unified plan of the municipal government tends to favor the development of Fenghua District in terms of allocation of public resources in particular. For example, the two higher education institutions that are under the jurisdiction of Fenghua District were established after the city-county merger policy was implemented.

**Fig 10:**
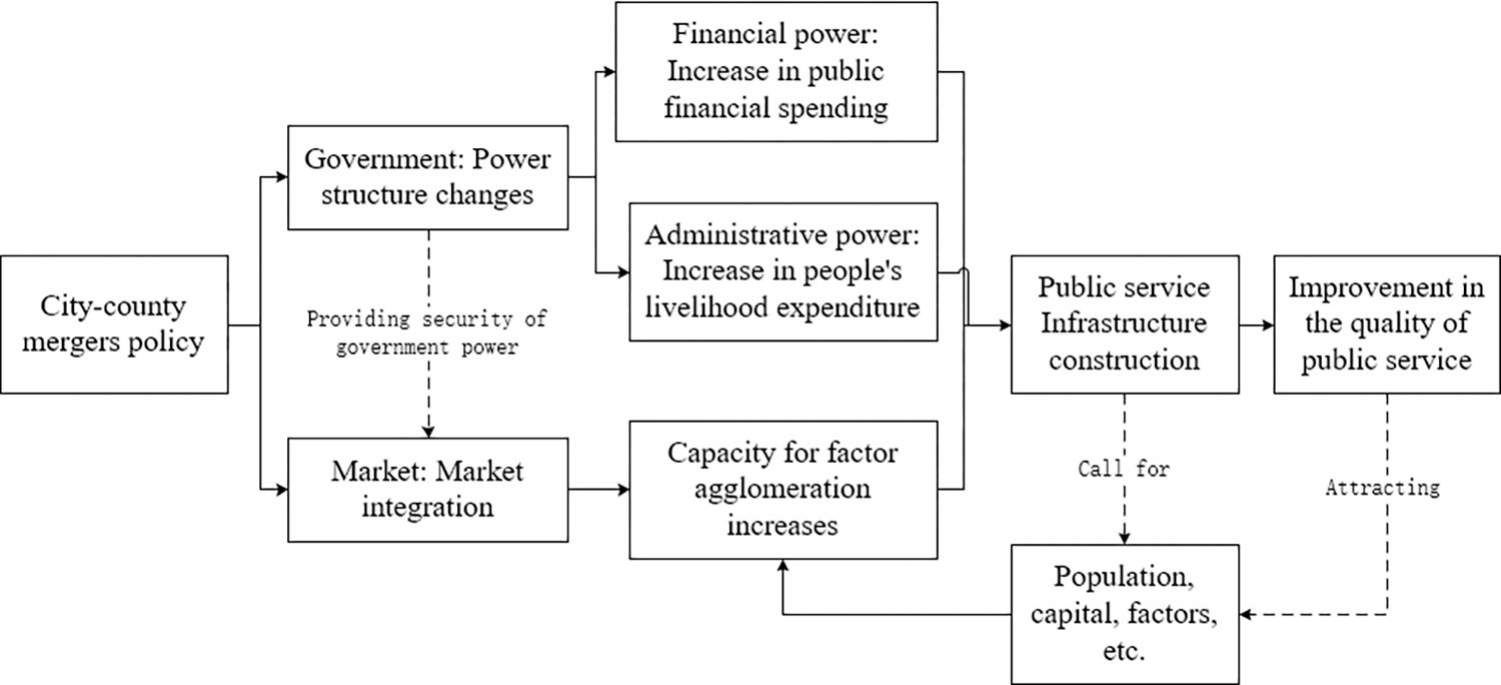
Transmission mechanism by which city-county mergers affect the quality of public services.

According to the previous theoretical analysis with the case study of Ningbo city, the transmission mechanism is proposed as shown in **Fig 10.** The left side of the figure represents the implementation of the city-county merger policy, the middle represents the transmission path, and the right represents the improvement of the quality of public services.

## Discussion and conclusion

Given that China has entered a new development phase since 2017, urban managers should improve the quality of public services to promote high-quality urbanization while continuously improving economic performance. Municipal amalgamation is one of the core policy tools for Chinese government intervention in urbanization, and the city-county mergers policy provides valuable research objects for examining whether government-led urban expansion improves the quality of public services. This paper presents a theoretical analysis and an empirical investigation of the policy effects and analyzes the transmission mechanisms linking city-county mergers and public services. Aiming to provide new insights into the study of municipal amalgamations, which will be meaningful for improving the quality of public services and promoting the new urbanization process.

We analyzed the policy effect between the policy of city-county mergers and public services based on city panel data at the municipal district level in China from 2003 to 2019 by the PSM-DID approach. The entropy method was used to measure the urban quality of public services. Measurements were made separately for education, health care, and transportation. The empirical results showed that the city-county mergers policy brought a 1% improvement in the quality of public services. The promotion effects of city-county mergers on education, medical care, and transportation were significant at the 1% level, with the largest influence on education. As a result of the transmission mechanism analysis in the case study of Fenghua District in Ningbo City, the theoretical analysis was further confirmed and refined. Under the strictly hierarchical administrative system in China, municipal agglomerations affected the quality of public services mainly through three paths, namely adjusting the government power structures and stimulating market integration, with the former including the allocation of financial power and administrative power. Further research showed that city-county mergers exhibit a negative correlation in terms of public services per capita, indicating that although the city-county mergers led to an improvement in the overall quality of urban public services, the issue of equalization of public services was still a prominent problem facing urban development.

Multiple tests were conducted to ensure the robustness of the results in the empirical study. In any case, the positive and significant policy effects may result from the change in statistical scope, with data from merged counties being included in the municipal districts. The empirical results were less convincing as a result. In our attempt to resolve this issue, we found that the large number of indicators involved in public services, as well as the usual lack of statistics in the yearbook regarding the merged counties, made data processing more difficult. However, based on the case study, we found that the data values of the merged counties were much smaller than those of the municipal districts and whether the data of the counties were summed up or not had little effect on the municipal districts. We therefore believed that the changes in data quality did not significantly affect the empirical findings. Additionally, the case study of Fenghua District in Ningbo City further confirmed the effect of the city-county merger on improving the quality of urban public services by using county data. We believe that our insightful analysis of the transmission mechanism will be a major contribution to existing research, and the empirical results still have some reference value.

As a result of our research, the following policy implications are proposed. First, city governments should make use of administrative restructuring policy tools appropriately to provide institutional guarantees from financial and administrative power allocations. The goal is to break down administrative barriers and promote regional integration, ultimately improving the quality of public services and optimizing the spatial distribution of public services. Second, city governments should invest more in public resources to foster a high-quality development environment. Public services should be focused not just on increasing their overall supply but also on improving their level of service per capita. It is particularly important to consider the optimal allocation of public resources among different categories other than education resources alone. Finally, the demand for public services from a large number of migrants in the process of urbanization should also be taken into account. City governments should continuously improve their dynamic adjustment mechanism to avoid unequal distribution of public resources and a loss of resource utilization efficiency.

Although certain institutional environments are unique to China, studying how administrative restructuring affects the quality of public services in China is still valuable for other developing countries. First, governments should pay attention to integrating public services to enhance the well-being of urban residents while promoting urbanization. Second, it is essential to maximize the allocation of public resources and promote the urban development process through institutional reforms such as municipal amalgamations. Lastly, governments should make improvements to the market supervision mechanisms, create a benign environment that promotes the free flow of factors in urban spaces, improving overall operational efficiency.

## Supporting information

S1 Table Data for descriptive statistics of variables. (XLSX)

S2 Table Data for quality of public services evaluation indexes. (XLSX)

S3 Table Time and cities for city-county mergers, 2003–2019. (XLSX)

S4 Table Government finance data for Ningbo City and Fenghua District. (XLSX)
